# Supplementary material for: Intratumoral Heterogeneity and Metabolic Cross-Feeding in a Three-Dimensional Breast Cancer Culture: An In Silico Perspective
Source: Int J Mol Sci. 2024 Oct 10;25(20):10894. doi: 10.3390/ijms252010894 (PMC11508025; doi:10.3390/ijms252010894)
Supplement: Supplementary file 1 [file ijms-25-10894-s001.zip › ijms-3192535-Supplementary Materials.pdf]

International Journal of *Molecular Sciences*

MDPI

Article

Intratumoral Heterogeneity and Metabolic Cross-Feeding in a Three-Dimensional Breast Cancer Culture: An In Silico Perspective

Jorge E. Arellano-Villavicencio [1,2], Aarón Vázquez-Jiménez [1], Juan José Oropeza-Valdez [3], Cristian Padron-Manrique [1,4], Heriberto Prado-García [5], Armando R. Tovar [6] and Osbaldo Resendis-Antonio [1,3,7,*]

1 Human Systems Biology Laboratory, Instituto Nacional de Medicina Genómica (INMEGEN), Mexico City 14610, Mexico; jorge.arellano.bioexp@gmail.com (J.E.A.-V.); vazqaaron@gmail.com (A.V.-J.); cristianjuliocesar.agualimpia@gmail.com (C.P.-M.)
2 Programa de Doctorado en Ciencias Bioquímicas, Universidad Nacional Autónoma de México (UNAM), Mexico City 04510, Mexico
3 Centro de Ciencias de la Complejidad, Universidad Nacional Autónoma de México (UNAM), Mexico City 04510, Mexico; juan.oropeza@c3.unam.mx
4 Programa de Doctorado en Ciencias Biomédicas, Universidad Nacional Autónoma de México (UNAM), Mexico City 04510, Mexico
5 Laboratorio de Onco-Inmunobiologia, Departamento de Enfermedades Crónico-Degenerativas, Instituto Nacional de Enfermedades Respiratorias Ismael Cosio Villegas (INER), Mexico City 14080, Mexico; hpradog@yahoo.com
6 Departamento de Fisiología de la Nutrición, Instituto Nacional de Ciencias Médicas y Nutrición Salvador Zubirán (INCMNSZ), Mexico City 14080, Mexico; armando.tovarp@incmnsz.mx
7 Coordinación de la Investigación Científica—Red de Apoyo a la Investigación, Universidad Nacional Autónoma de México (UNAM), Mexico City 14080, Mexico
* Correspondence: oresendis@inmegen.gob.mx

check for updates

Citation: Arellano-Villavicencio, J.E.; Vázquez-Jiménez, A.; Oropeza-Valdez, J.J.; Padron-Manrique, C.; Prado-García, H.; Tovar, A.R.; Resendis-Antonio, O. Intratumoral Heterogeneity and Metabolic Cross-Feeding in a Three-Dimensional Breast Cancer Culture: An In Silico Perspective. *Int. J. Mol. Sci.* **2024**, *25*, 10894. https://doi.org/10.3390/ijms252010894

Academic Editor: Aaron C. Tan

Received: 20 August 2024
Revised: 2 October 2024
Accepted: 8 October 2024
Published: 10 October 2024

Copyright: © 2024 by the authors. Licensee MDPI, Basel, Switzerland. This article is an open access article distributed under the terms and conditions of the Creative Commons Attribution (CC BY) license (https://creativecommons.org/licenses/by/4.0/).

Supplementary

1. Growing Medium

Table S1. Culture medium. The culture medium used for the reconstructions was based on Leibovitz L-15 Medium (ATCC 30-2008). More information on the medium can be found at https://www.atcc.org/products/30-2008.

Inorganic Salts	Amount (g/L)	Amino Acids	Amount (g/L)
$CaCl_2$ (anhydrous)	0.14	L-Alanine	0.225
$MgCl_2{\cdot}6H_2O$	0.2	L-Arginine (free base)	0.5
$MgSO_4$ (anhydrous)	0.09767	L-Asparagine·H_2O	0.25
KCl	0.4	L-Cysteine (free base)	0.12
KH_2PO_4 (anhydrous)	0.06	L-Glutamine	0.3
NaCl	8.0	Glycine	0.2
Na_2HPO_4 (anhydrous)	0.19	L-Histidine (free base)	0.25
		L-Isoleucine	0.125
		L-Leucine	0.125
		L-Lysine·HCl	0.0937

Table S1. *Cont.*

Inorganic Salts	Amount (g/L)	Amino Acids	Amount (g/L)
		L-Methionine	0.075
		L-Phenylalanine	0.125
		L-Serine	0.2
		L-Threonine	0.3
		L-Tryptophan	0.02
		L-Tyrosine·2Na·$2H_2O$	0.43
		L-Valine	0.1
**Vitamins**	**Amount (g/L)**	**Others**	**Amount (g/L)**
Choline Chloride	0.001	D-Galactose	0.9
Riboflavin-5·PO_4·Na·$2H_2O$	0.001	Phenol Red, Sodium Salt	0.01
Folic Acid	0.001	Sodium Pyruvate	0.55
myo-Inositol	0.002		
Nicotinamide	0.001		
D-Pantothenic Acid (hemicalcium)	0.001		
Pyridoxine·HCl	0.001		
Thiamine·PO_4·Cl·$2H_2O$	0.001		

2. Characteristics of the Metabolic Reconstructions

Table S2. Characteristics of the metabolic reconstructions. Included are the count of metabolic reactions within each GEM, as well as the number of metabolites within each of these classified as follows: I—invasive, R—reservoir, and P—proliferative. For more information on each reconstruction, consult the MEMOTE reports.

Subpopulation	Relative Abundance		Reactions	Metabolites
	Day 6	Day 19		
**Invasive**	0.0977	0.5739	1126	982
**Reservoir**	0.391	0.2739	742	616
**Proliferative**	0.5113	0.1522	1032	807

3. Output Files of Community Simulation with MICOM

Supplementary File S1 contains 4 tables of MICOM output files:

- Community fluxes on day 6 (Day6_fluxes);
- Community fluxes on Day 19 (Day19_fluxes);
- Exchanges fluxes with the medium on day 6 (Exchanges_day6);
- Exchange fluxes with the medium on day 19 (Exchanges_day19).

Download the files in the Github MICOM/Output_files/csv_files folder: https://github.com/resendislab/Modeling_Heterogeneity_Cancer_Metabolism_MICOM

4. Reports of Quality with MEMOTE

Supplementary File S2—Report of MEMOTE (Invasive.html)
Supplementary File S3—Report of MEMOTE (Reservoir.html)
Supplementary File S4—Report of MEMOTE (Proliferative.html)
Download the files in the Github MICOM/Output_files/Reports_of_quality folder: https://github.com/resendislab/Modeling_Heterogeneity_Cancer_Metabolism_MICOM.

5. Enrichment of Metabolic Pathways with Active Reactions

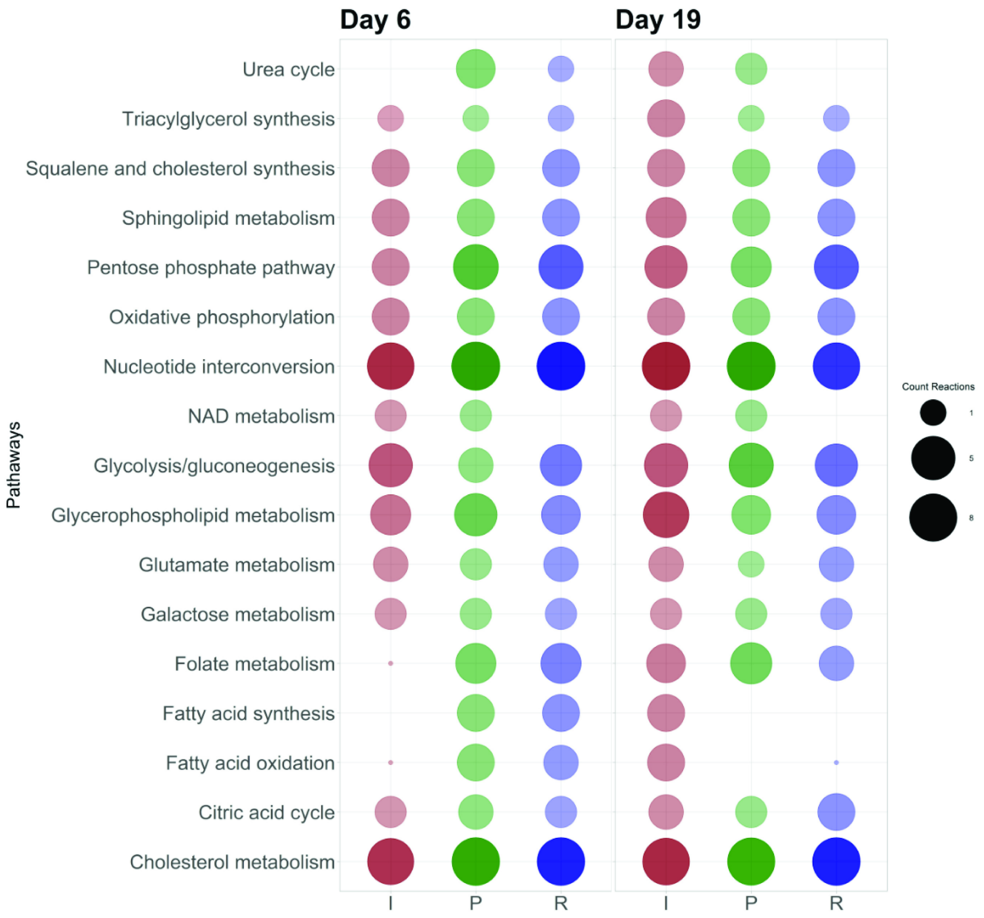

Figure S1. Number of reactions with fluxes per metabolic pathway. The size of the spheres corresponds to the number of active reactions within each pathway for the 3 subpopulations. A blank space indicates that the reactions in this case had fluxes equal to zero, a reason for discarding. This behavior differs between days 6 and 19, and it can be deduced that, depending on the day, the metabolism is reconfigured; different metabolic behaviors are observed between the subpopulations depending on the day of the study.

6. Intracellular Reactions with Activity (Reaction Directionality)

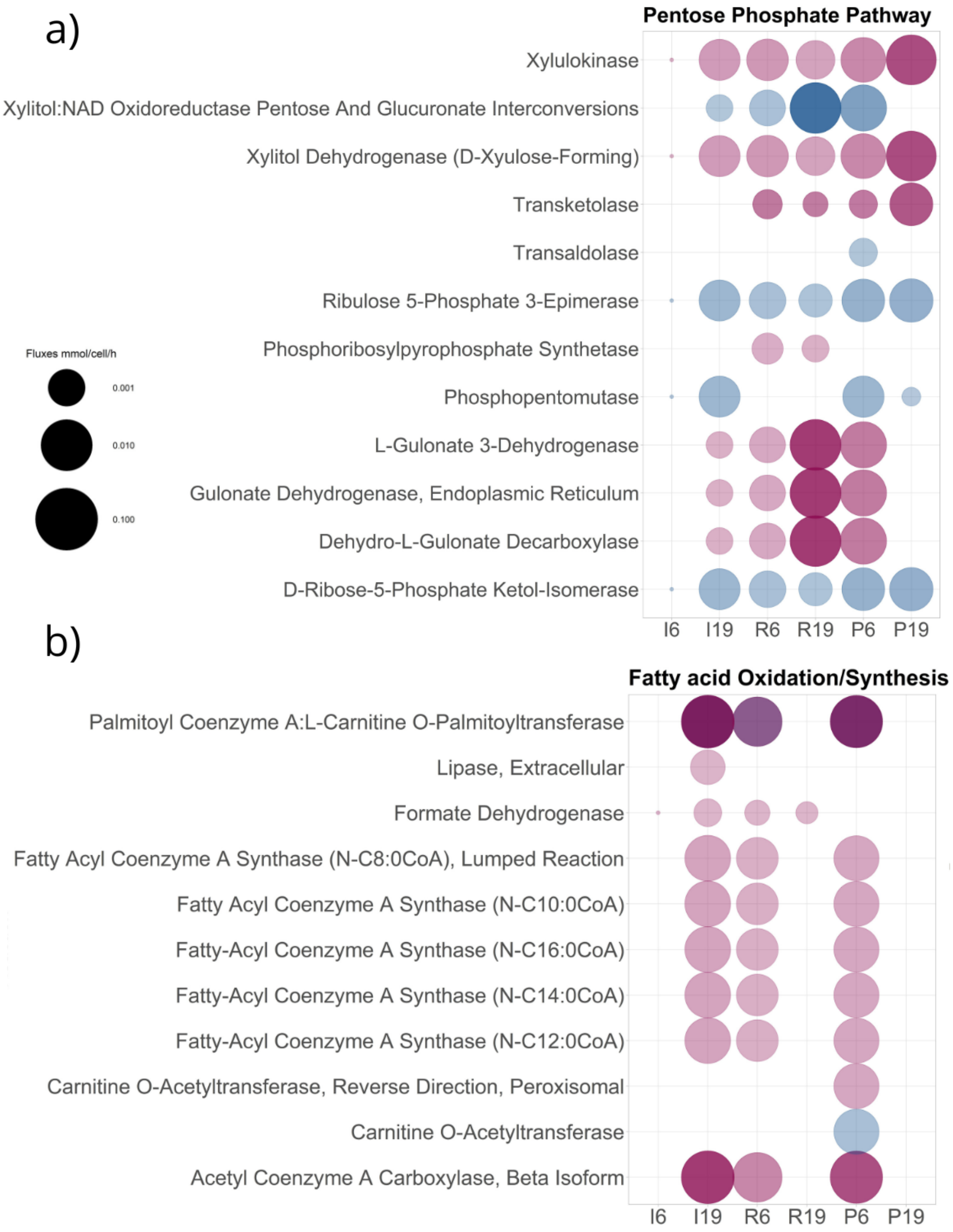

Figure S2. Enzymes with metabolic activity. The metabolic pathways were grouped according to the VMH database (see Section 5) in which each reaction is assigned a subsystem such as (**a**) pentose phosphate pathway and (**b**) fatty acid oxidation/synthesis. Only reactions with activity in at least one of the three subpopulations were considered for visualization. To represent the directionality, a color identifier was used: the reddish color corresponds to the directionality of the reaction in its classical pathway described in the database, and blue corresponds to a reversal of the directionality of the reaction when observed. The blanks do not exclude the presence of enzymes in the reconstruction; they only reflect whether there was activity. The units of the FBA modeling correspond to the metabolic biotransformation rate (mmol/cell/hour) reflected in the size of the spheres for each reaction.

7. Community Response to Oxygen Gradients

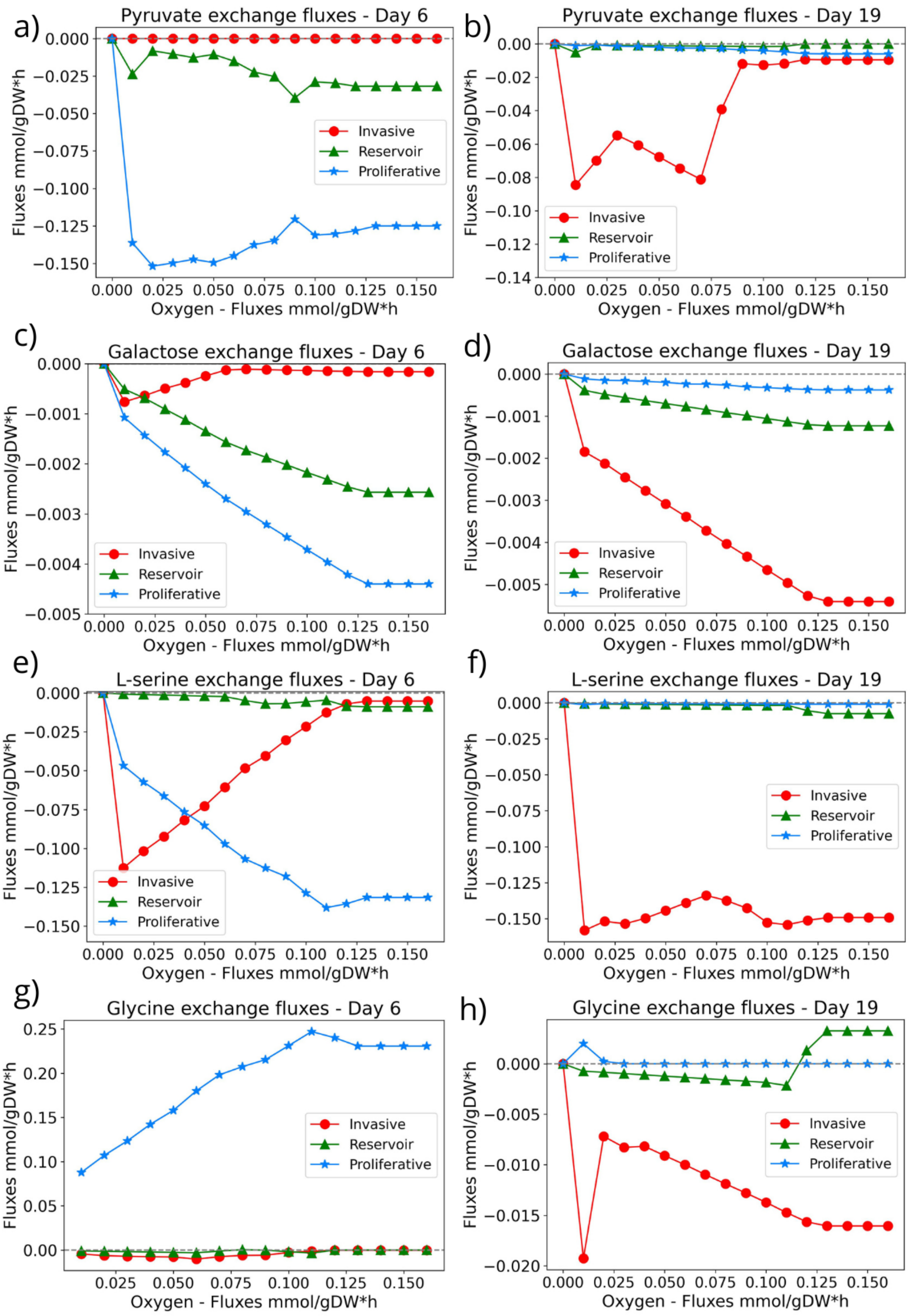

Figure S3. Community response to oxygen depletion. From right to left, the gradient from normoxia to hypoxia is represented, with oxygen fluxes decreasing, and each of the 16 marked points reflects the behavior of the subpopulations in terms of the consumption or production of a metabolite. Values above zero represent secretion, and those below zero represent consumption. Red lines correspond to invading cells, green lines to reservoirs, and blue lines to proliferative cells. Each point on the X-axis represents a different community simulation or scenario. As this is a deterministic scenario, we do not have any statistics since there are no variations in the repetitions of the simulations. To visualize the behavior of the community with the disposition of the elements of the medium, we selected metabolites that could be used as an energy source on different study days: (**a**) pyruvate, (**c**) galactose, (**e**) L-serine, and (**g**) lysine on day six and (**b**) pyruvate, (**d**) galactose, (**f**) L-serine, and (**h**) glycine on day 19.

8. Doubling Times

According to Meadows et al., the doubling time results from dividing the natural logarithm of 2 by the biomass or growth rate. The simulation indicated a 0.026 mmol/cell/h rate, corresponding to an approximate 26 h doubling time on both days. The doubling time is a characteristic unit (a natural unit of scale) for the exponential growth equation, and its reciprocal for exponential decay is the half-life [1].

$$\text{Doubling Time} = \frac{\ln 2}{\text{Biomass}}$$

9. Robustness Tests

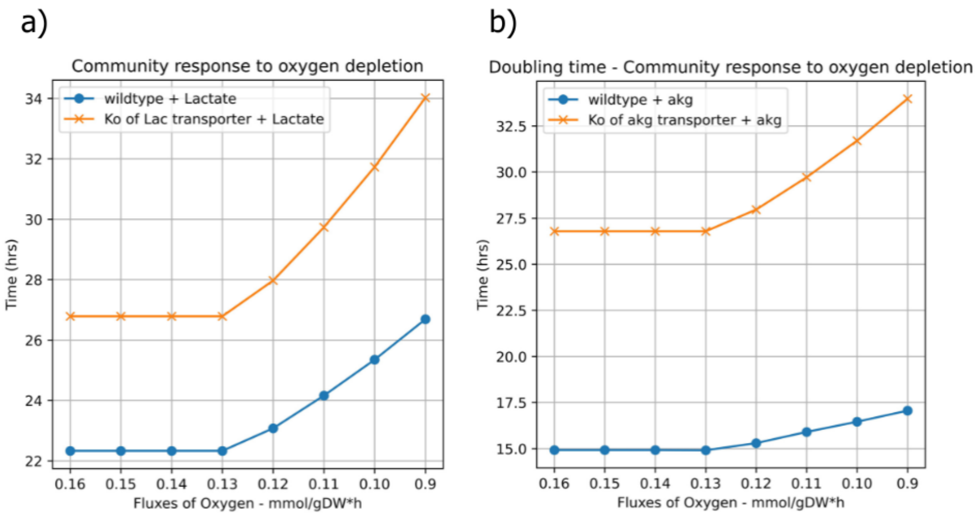

Figure S4. Response to the addition of external carbon sources in the community. (**a**) Addition of lactate in the medium vs. KO of lactate transporters. The blue line represents the behavior of the MCF7 community under an oxygen gradient with lactate in the medium. At low levels, the doubling times are close to a standard condition of 26 h. The opposite is the case with a blockade of lactate transporters, indicated by the orange line. (**b**) Addition of AKG in the medium vs. KO of AKG transporters. Doubling times with AKG decrease even more than with lactate, and blocking transport mitigates the effect. Also, in response to the oxygen gradient, there was no effect when AKG was added to the culture medium.

Reference

1. Meadows, D.H. *Thinking in Systems: A Primer*; Chealsea Green Publising: White River Junction, VT, USA, 2008.
