# Supplementary figures and images for: Intratumoral Heterogeneity and Metabolic Cross-Feeding in a Three-Dimensional Breast Cancer Culture: An In Silico Perspective
Source: Int J Mol Sci. 2024 Oct 10;25(20):10894. doi: 10.3390/ijms252010894 (PMC11508025; doi:10.3390/ijms252010894)

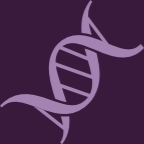

# International Journal of *Molecular Sciences*

Supplement: Supplementary file 1 [file ijms-25-10894-s001.zip › Definitions/ijms-logo-eps-converted-to.pdf]

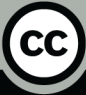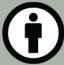

BY

Supplement: Supplementary file 1 [file ijms-25-10894-s001.zip › Definitions/logo-ccby-eps-converted-to.pdf]

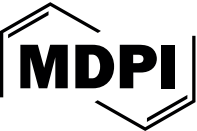

Supplement: Supplementary file 1 [file ijms-25-10894-s001.zip › Definitions/logo-mdpi-eps-converted-to.pdf]

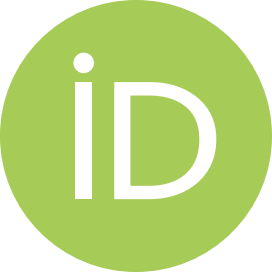

Supplement: Supplementary file 1 [file ijms-25-10894-s001.zip › Definitions/logo-orcid.pdf]

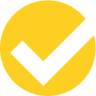

check for  
updates

Supplement: Supplementary file 1 [file ijms-25-10894-s001.zip › Definitions/logo-updates-eps-converted-to.pdf]

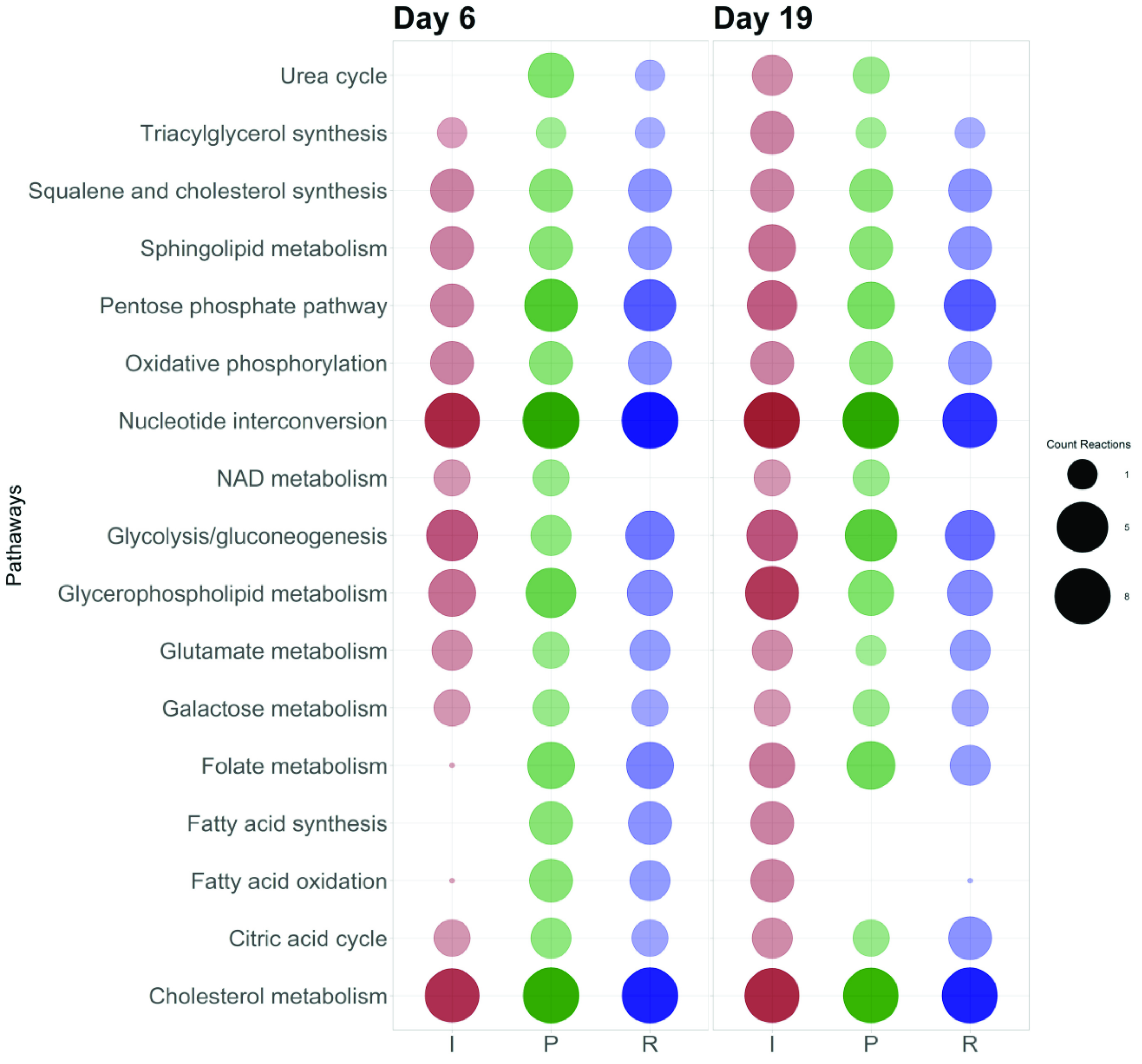

Supplement: Supplementary file 1 [file ijms-25-10894-s001.zip › SFigure 1.png]

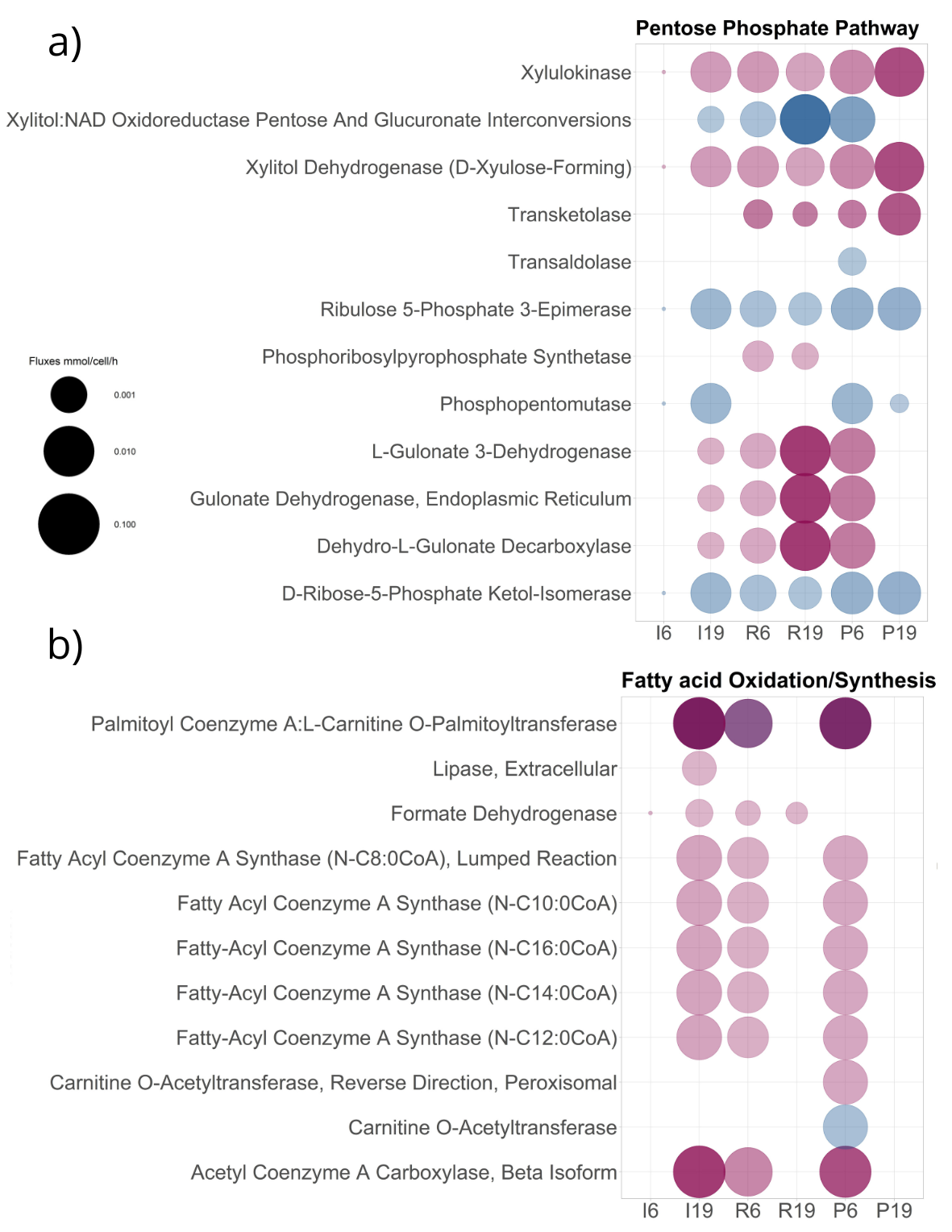

Supplement: Supplementary file 1 [file ijms-25-10894-s001.zip › SFigure 2.png]

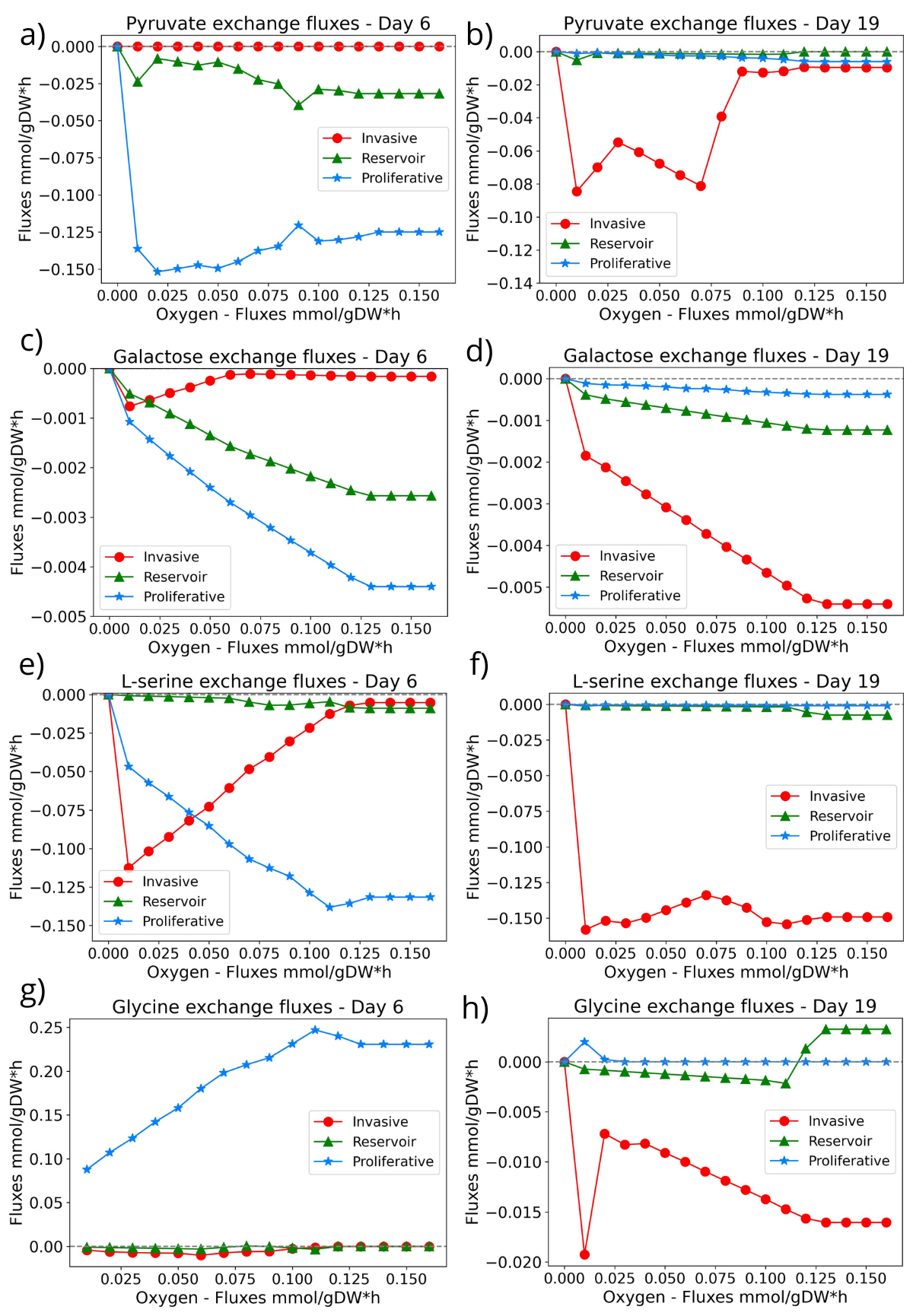

Supplement: Supplementary file 1 [file ijms-25-10894-s001.zip › SFigure 3.png]

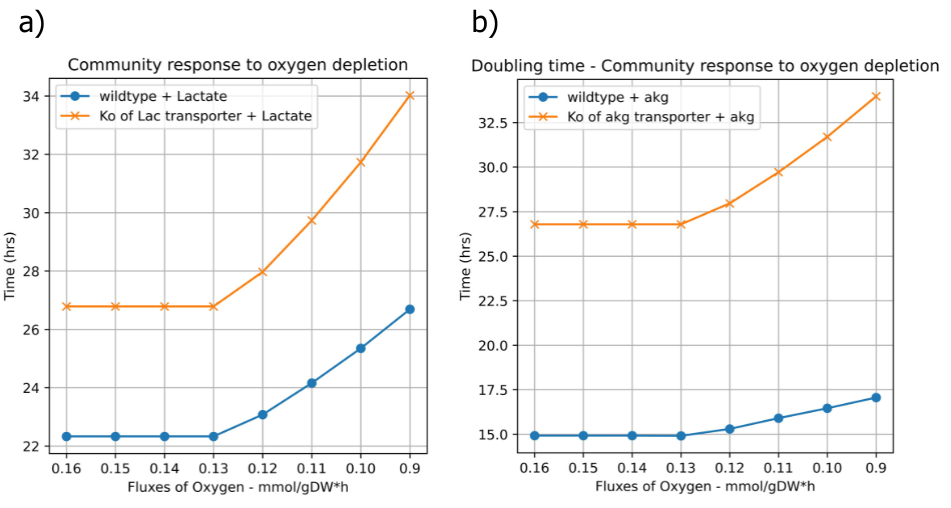

Supplement: Supplementary file 1 [file ijms-25-10894-s001.zip › SFigure 4.png]
